# Solvent Hydrogen Bonding Determines Adsorption Geometry of Trimesic Acid on Cu

**DOI:** 10.1002/anie.3523377

**Published:** 2026-07-29

**Authors:** Anusha Lalitha, Qixuan Li, Marialore Sulpizi, Denis Andrienko, Katrin F. Domke

**Affiliations:** ^1^ Institut Charles Gerhardt Montpellier (UMR CNRS 5253) Université Montpellier Montpellier France; ^2^ Faculty of Physics and Astronomy Ruhr University Bochum Bochum Germany; ^3^ Max Planck Institute for Polymer Research Mainz Germany; ^4^ Physical Chemistry II University of Duisburg‐Essen Essen Germany; ^5^ Present address: Janssen Research and Development Janssen Pharmaceutica N. V. Beerse Belgium

**Keywords:** molecular adsorption geometry, molecular dynamics simulations, solid/liquid interfaces, surface spectroscopy, tip‐enhanced Raman spectroscopy

## Abstract

The adsorption geometry of functional molecules largely determines the interfacial behaviour in various applications like catalysis, photovoltaics and molecular electronics. Understanding adsorbate orientation has remained a challenge due to lack of suitable in situ methods, thus hindering controlled device behaviour. Here, a combined tip‐enhanced Raman spectroscopy, density functional theory and molecular dynamics simulation approach elucidates the prevailing controversy about the adsorption geometry of showcase trimesic acid, one of the most widely used functional organic linkers, on Cu. We find that subtle energy differences in adsorbate–substrate–solvent interactions on the scale of 0.25 eV are decisive for the molecular orientation at solid/liquid versus solid/gas interfaces, emphasising the strength of combined theoretical and experimental quantitative in situ nanoscale characterisation for the rational design of functional molecular interfaces.

## Graphical abstract

Water reorients adsorbate molecules. Combined tip‐enhanced Raman spectroscopy, density functional theory and molecular dynamics simulations reveal the adsorption geometry of trimesic acid on Cu. Subtle adsorbate–substrate–solvent energy differences due to hydrogen bonding dictate molecular orientation at solid/liquid and solid/gas interfaces, providing a foundation for the rational design of functional molecular interfaces.

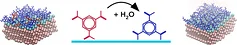

## 1 Introduction

Molecular adsorption geometry at surfaces often dictates chemical or charge transfer behaviour, for example, in catalysis [1, 2], photovoltaics [3, 4], molecular electronics [5], or material self‐assembly [6]. The adsorption geometry is governed by a delicate balance of the interactions between molecules, substrate and, in the case of a liquid phase, solvent species. To achieve improved device design, where molecular adsorption is predictable and strategically controlled, an accurate nanoscale description of the anchoring of organic molecules to solid surfaces under realistic working conditions is essential but can be rather difficult to achieve.

Particularly, chemical insights into site‐specific adsorption properties (as opposed to ensemble average responses) at solid/liquid interfaces have been difficult to obtain due to the limited availability of suitable in situ nanoscopy tools. Due to the presence of the solvent, molecules at solid–liquid interfaces often display a different behaviour from molecules at solid–gas interfaces [7, 8]. As the various molecule–substrate–solvent interaction energies are difficult to quantify, the origin of discrepancies in adsorption behaviour in different environments is difficult to elucidate.

One of the most widely employed organic linkers for functional materials and a showcase example for carboxylic acid anchoring groups omnipresent, for example, in photovoltaics and molecular electronics, is benzene tricarboxylic acid (or trimesic acid, TMA). The adsorption interactions of TMA‐metal have been extensively studied in ultrahigh vacuum, ambient gas phase and aqueous environments. From scanning tunnelling microscopy images, vibrational and photoelectron spectroscopies, flat [9], tilted [10, 11] or upright monopodal [9, 12] or bipodal [9, 13] adsorption geometries at the same metal surface (Cu) have been concluded.

To understand and quantify the physico‐chemical interactions that govern linker orientation, it is necessary to conduct comparable gas‐ and liquid‐phase experiments that provide molecular‐level resolution probing uniform adsorption sites and to quantify the energetic balances between linker–surface, linker–linker and linker–solvent interactions with the help of advanced simulation tools. Such insights into the subtle energy balance of self‐assembly would greatly aid controlled molecular surface anchoring for strategic device design.

A powerful experimental tool to investigate molecular scale behaviour at solid/gas and solid/liquid interfaces is tip‐enhanced Raman spectroscopy (TERS) [14, 15]. A scanning‐probe based nearfield Raman approach, TERS provides nanoscale chemical spatial resolution and sufficient sensitivity to obtain vibrational fingerprints of very few or even single molecules at a time that contain information about the molecular interactions at a surface. TER spectra can be analysed like conventional Raman spectra in terms of the presence or absence of vibrational modes, relative band intensities and frequency shifts to precisely determine the molecular adsorption geometry [16, 17].

To interpret the experimental results on molecular adsorption geometry and solvent effects, it is necessary to build a model structure of the adsorbed layer and validate it. At this point, computer simulations have proven to be helpful by providing explicit information at an atomic level of resolution. Typically, multi‐scale approaches are used, incorporating simulations from ab initio to atomistic to coarse‐grained levels of system description [18, 19]. To this end, simulations have been extensively used to elucidate solvent effects on molecular adsorption on various substrates including Cu [20, 21, 22, 23].

Here, we study the adsorption behaviour of TMA on a Cu/Au(111) surface in gas and in liquid phase to elucidate the surface binding and orientation‐determining parameters for widely employed carboxyl linker molecules. In correlation with density functional theory (DFT) results, TER spectra show that molecules reorient from a mixed mono‐ and bipodal adsorption configuration to a favoured bipodal geometry in the condensed phase. We explain the observed rearrangement by quantifying the energy difference due to TMA hydrogen bonding with water solvent molecules with molecular dynamics (MD) simulations. Our uniquely precise insight gained with the powerful combination of TERS/DFT and MD explains the controversy about TMA adsorption at Cu found in literature and underlines the general importance of studying reactive interfaces under realistic conditions, as adsorbate behaviour in gas‐phase can differ substantially from the one in solvent.

**FIGURE 1:**
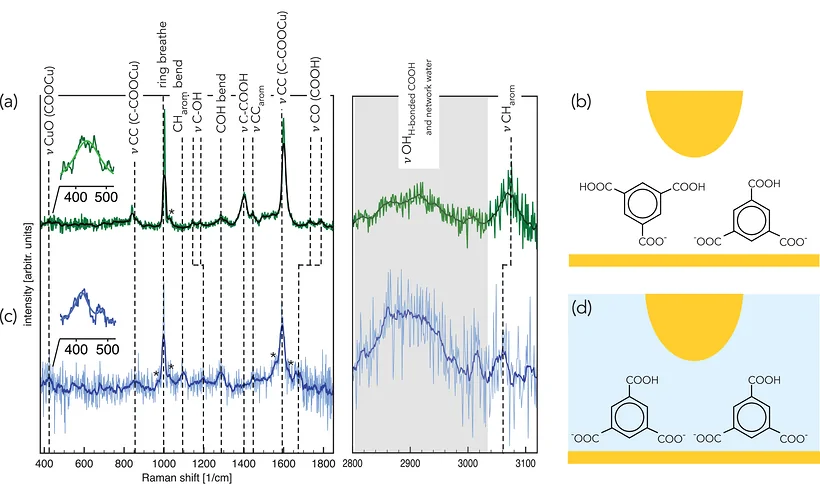
Average TER spectra of TMA at ${\mathrm{Cu}}_{UPD}$/Au(111) recorded in Ar (a) and in $H_{2}O$ (c) with sample bias, $E_{bias}$ = 0.1 V and tunnelling current, $I_{t}$ = 0.1 nA (Ar) and 0.2 nA ($H_{2}O$). The spectra are normalised to the peak area of the peak at ca. 1000 ${\mathrm{cm}}^{-1}$ and are y‐offset for clarity. Insets are zooms of the 400 ${\mathrm{cm}}^{-1}$‐region. Bands denoted with an asterisk are assigned to bending/scissoring modes of TMA‐network coordinated $H_{2}O$. (b) and (d) depict the adsorbate geometries in Ar and in $H_{2}O$, respectively.

Figure 1 shows the average TER spectra (background corrected according to the procedure proposed by Ren and coworkers) [24] of a monolayer of TMA adsorbed on an underpotentially deposited Cu adlayer, ${\mathrm{Cu}}_{UPD}$, on Au(111) recorded in Ar (a, green) and in water (c, blue), respectively. The raw data (light) were smoothened (dark) and then normalised to the intensity of the ring breathing mode of TMA at ca. 1000 ${\mathrm{cm}}^{-1}$, which remains essentially unaffected by TMA‐Cu, TMA‐TMA or TMA‐$H_{2}O$ interactions according to our DFT results (Supporting Information). The TER spectra were recorded on a ca. 200 nm wide, defect‐free terrace (as confirmed by STM imaging prior to TERS experiments, Supporting Information) to ensure probing homogeneous adsorption sites of the molecules. Typically, the spatial resolution of ambient TERS for non‐resonant molecules in a Au–Au tip‐sample gap is in the order of a few nanometers [25, 26]; conservatively assuming an effective TERS scattering diameter of 5 nm and a TMA closest packing of 2 molecules per ${\mathrm{nm}}^{2}$ [13], we probe around 40 TMA molecules.

In general, the TER spectra collected in Ar show a signal‐to‐noise ratio that is approximately three times larger than the one of in‐water experiments, in agreement with previous reports about the setup sensitivity [27]. We have assigned the bands with the help of DFT‐based Raman frequencies and tensors (Supporting Information). In brief, to model Raman spectra in an inert atmosphere and in water, we adopted four model geometries, depending on the bonding pattern of TMA on Cu. These geometries are shown in Figure S12. The model geometries were first energy‐optimised at the cam‐b3lyp/6‐311g(d,p) level of theory with a subsequent calculation of (polarised) Raman spectra. The combined TERS and DFT data consistently point to the fact that TMA is deprotonated during sample preparation and binds either with one (monopodal) or with two carboxylate groups (bipodal) to the Cu adlayer when exposed to Ar (Figure 1b) but shows a preferential bipodal adsorption in $H_{2}O$ (Figure 1d) as discussed in the following.

The covalent binding of TMA with the Cu surface is evidenced by the appearance of a low‐frequency Cu‐OOC stretch mode. The appearance of two clear peaks at 417 and 480 ${\mathrm{cm}}^{-1}$ in water is consistent with a bipodal adsorption configuration according to DFT. The weak, broad feature observed in Ar at 437 ${\mathrm{cm}}^{-1}$ is in line with a less ordered, mixed monopodal and bipodal adsorption geometry. Band broadening is typically a result of various surface atoms or linker groups being involved in forming a metal–adsorbate complex. Regarding the ν CC ($C_{arom}$‐COOCu) stretch mode, DFT results predict two (one) ν(OOC‐$C_{arom}$) modes for a monopodal (bipodal) geometry. Therefore, we assign the sharp feature at 841 ${\mathrm{cm}}^{-1}$ with a shoulder at 869 ${\mathrm{cm}}^{-1}$ in Ar to the simultaneous presence of TMA bound with one or two carboxylate groups and the single broad feature present in water at 863 ${\mathrm{cm}}^{-1}$ to bipodally adsorbed TMA.

All spectra show a prominent CC ring breathing mode around 1000 ${\mathrm{cm}}^{-1}$, indicating an upright TMA adsorption geometry where the benzene ring plane, and thus the *x,y*‐elements of the Raman polarisability tensor, are oriented parallel to the surface normal (i.e., the nearfield lines in the tip‐sample gap), according to TERS selection rules [28]. DFT calculations result in a nearly identical frequency of 1002/3 ${\mathrm{cm}}^{-1}$ for the ring breathing modes of the various TMA systems explored—with one exception of the bipodal TMA complex in water that has a slightly lower Raman shift of 996 ${\mathrm{cm}}^{-1}$. As such, apparently, the ring stretching frequency is sensitive to the number of anchoring groups, but rather independent of the chemical environment of the TMA molecule. The calculated frequencies are surprisingly close to the TERS ones, that is, 1002 ${\mathrm{cm}}^{-1}$ in Ar and 999 ${\mathrm{cm}}^{-1}$ in $H_{2}O$, and further support the conclusion that TMA adsorbs in a bipodal fashion at the solid/liquid interface.

Similarly, the appearance of the aromatic CH bending mode (1098 ${\mathrm{cm}}^{-1}$) solely in water is a clear indication of bipodal adsorption in water, given the fact that DFT predicts only negligible CH bending intensity for monopodal Cu–OOC interaction, but a six times stronger one for a bipodal configuration in the presence of $H_{2}O$ (Supporting Information). Note that the experimental Raman shift of 1098 ${\mathrm{cm}}^{-1}$ is about 140 ${\mathrm{cm}}^{-1}$ lower than the calculated one for hydrated 2‐Cu TMA of 1245 ${\mathrm{cm}}^{-1}$, albeit close to the one for fully hydrated TMA of 1106/8 ${\mathrm{cm}}^{-1}$, which points toward a strong network‐type interaction between TMA and $H_{2}O$ molecules.

Interestingly, the TERS‐in‐water spectrum exhibits small shoulder peaks at 970 and 1027 ${\mathrm{cm}}^{-1}$ as well as at 1560 and 1623 ${\mathrm{cm}}^{-1}$, denoted with asterisks in Figure 1, that, according to the DFT results, can be assigned to H‐bonded $H_{2}O$ bending and scissoring modes, respectively (Supporting Information). Note that we have never observed clear modes in TERS experiments in pure water of water molecules confined to the interface or localised in the tip‐sample gap. The farfield signal of bulk water typically masks all potential small nearfield signals from interfacial water molecules. Only in the data at hand where TMA is previously adsorbed on the surface do we observe these modes. Thus, we speculate that the detected H‐bonded water molecules must form part of the TMA adsorbate structure or network [29, 30], which in turn enhances the $H_{2}O$ polarisability and thus the scattering cross section sufficiently to enable TERS detection. It is known that $H_{2}O$ bending and stretching modes can be largely enhanced in hydrogen‐bonded anionic complexes due to photon‐induced charge transfer [31] and/or strong polarisation effects [32], which can be achieved, for example, through co‐adsorption of (pseudo‐)halide ions or by applying very negative electrode potentials. Given the observed drastic shifts of specific TMA/Cu modes in water compared to in Ar and the clear presence of $H_{2}O$ bending and scissoring modes in the TMA TER spectra, the formation of a TMA–water network with partial charge transfer seems to be a likely scenario in the present case. Note that for TERS in Ar, we also observe one small shoulder peak at 1035 ${\mathrm{cm}}^{-1}$ that might be explained by tightly bound (network) water molecules left after incomplete water evaporation during sample preparation.

The TER spectra exhibit several bands associated with the COH moiety of the COOH groups: in accordance with DFT, for the C–OH stretch mode, a double peak with maxima at 1146 and 1181 ${\mathrm{cm}}^{-1}$ is found in gaseous environment in line with a mixed monopodal and bipodal adsorption, whereas only one peak at a slightly larger Raman shift of 1198 ${\mathrm{cm}}^{-1}$ is found in water for bipodal geometry. While the frequency of the COH bending mode is similar in both environments (1290 and 1286 ${\mathrm{cm}}^{-1}$), indicating comparable TMA‐TMA and/or TMA‐$H_{2}O$ H‐bond interactions), the peak is much more pronounced in water than in Ar relative to the ring breathing band. As similarly weak intensities are found for 1‐Cu and 2‐Cu TMA calculated modes in air, but more than twice the intensity is predicted for 2‐Cu compared to 1‐Cu in water, the DFT and TERS results are consistent with the above‐discussed adsorption geometries.

The strong TER band at 1403 ${\mathrm{cm}}^{-1}$ that is only visible in Ar is assigned to the C‐COOH stretch mode of a “free” carboxyl group, namely, the only mode present in DFT gas phase spectra in the absence of $H_{2}O$ at 1345 ${\mathrm{cm}}^{-1}$ for 1‐Cu and 2‐Cu TMA. When TMA is hydrated, the C‐COOH stretch blueshifts and overlaps with C═$C_{arom}$ and further C‐COOCu stretch modes in the region between 1450 and 1600 ${\mathrm{cm}}^{-1}$ (in DFT and TERS).

Another vibrational mode sensitive to the adsorption geometry and the environment is the C═O stretch of the carboxyl(ate)s groups. The one TERS peak of medium intensity recorded in $H_{2}O$ at 1673 ${\mathrm{cm}}^{-1}$ is redshifted by about 60–110 ${\mathrm{cm}}^{-1}$ compared to the two weak peaks found in Ar at 1734 and 1787 ${\mathrm{cm}}^{-1}$. According to the DFT results, this difference in Raman shift can be explained by the H‐bond strength in TMA–TMA and TMA–$H_{2}O$ interactions. In excellent agreement, for TMA–TMA interactions, the ν(C═O) is predicted by DFT to redshift by 60–110 ${\mathrm{cm}}^{-1}$ compared to the “free” COOH for the TMA tetramer structure. Upon the addition of water, DFT predicts a redshift of 70 ${\mathrm{cm}}^{-1}$ compared to the “free” COOH group. As such, the observations for the ν(C═O) mode are yet another clear indicator for a mixed adsorption in Ar and a bipodal TMA orientation in water.

In the upper spectral region between 2800 and 3200 ${\mathrm{cm}}^{-1}$, we find OH and aromatic CH stretch modes. The OH stretch modes are located at rather low frequencies between ca. 2800 and 3030 ${\mathrm{cm}}^{-1}$ in the TER spectra. This observation is in accordance with the DFT results that predict a significant redshift of the OH stretch frequency of the COOH moeity to 2860–3120 ${\mathrm{cm}}^{-1}$ when it is H‐bonded to either other TMA molecules or to $H_{2}O$ molecules compared to the “free” COO‐H stretch (at ca. 3500 ${\mathrm{cm}}^{-1}$). Similar stretching frequencies of H‐bonded carboxyl‐OH modes between 2500 and 3000 ${\mathrm{cm}}^{-1}$ have been repeatedly reported in the literature [33, 34].

The TERS aromatic CH stretch modes appear at 3071 and 3059 ${\mathrm{cm}}^{-1}$ in Ar and $H_{2}O$, respectively. Note here that the full‐width‐half‐maximum of the in‐Ar peak (fwhm = 43) is more than twice as large as the one of the in‐water peak (fwhm = 16), indicating a less ordered adsorption of TMA in Ar. The difference in Raman shift is in excellent agreement with the DFT results that predict a redshift of 12–18 ${\mathrm{cm}}^{-1}$ for bipodal adsorption in $H_{2}O$ compared to the monopodal/$H_{2}O$ and the in‐vacuum systems. Hence, the differences in Raman shift and fwhm of the ν(${\mathrm{CH}}_{arom}$) modes fully support the conclusion that TMA is adsorbed with two carboxylate groups at Cu in water and in a mixed orientation in Ar.

Furthermore, we can employ the respective ${\mathrm{OH}}_{H-bonded}$/CH stretch intensity ratios as obtained from Lorentzian band fits (Supporting Information), $I_{OH/CH}$, to quantify the relative number of TMA Hbonds in Ar compared to in water. This ratio is a factor 6.5 larger in $H_{2}O$ ($I_{OH/CH}$ = 16.4) than in Ar ($I_{OH/CH}$ = 2.5), which can be explained by a, respectively, larger number of H‐bonds formed in the presence of (network) $H_{2}O$ molecules compared to the predominant TMA–TMA H‐bond formation (with negligible amounts of residual water) in Ar.

To summarise the first part, TERS and DFT results consistently point to the picture that the TMA molecules are adsorbed to the Cu/Au(111) surface through one or two deprotonated carboxylate bonds in Ar (or in air) while in the presence of water TMA favours bipodal adsorption, for the given adsorption procedure. How can we explain this different adsorption geometry in the presence of water compared to gas phase?

**FIGURE 2:**
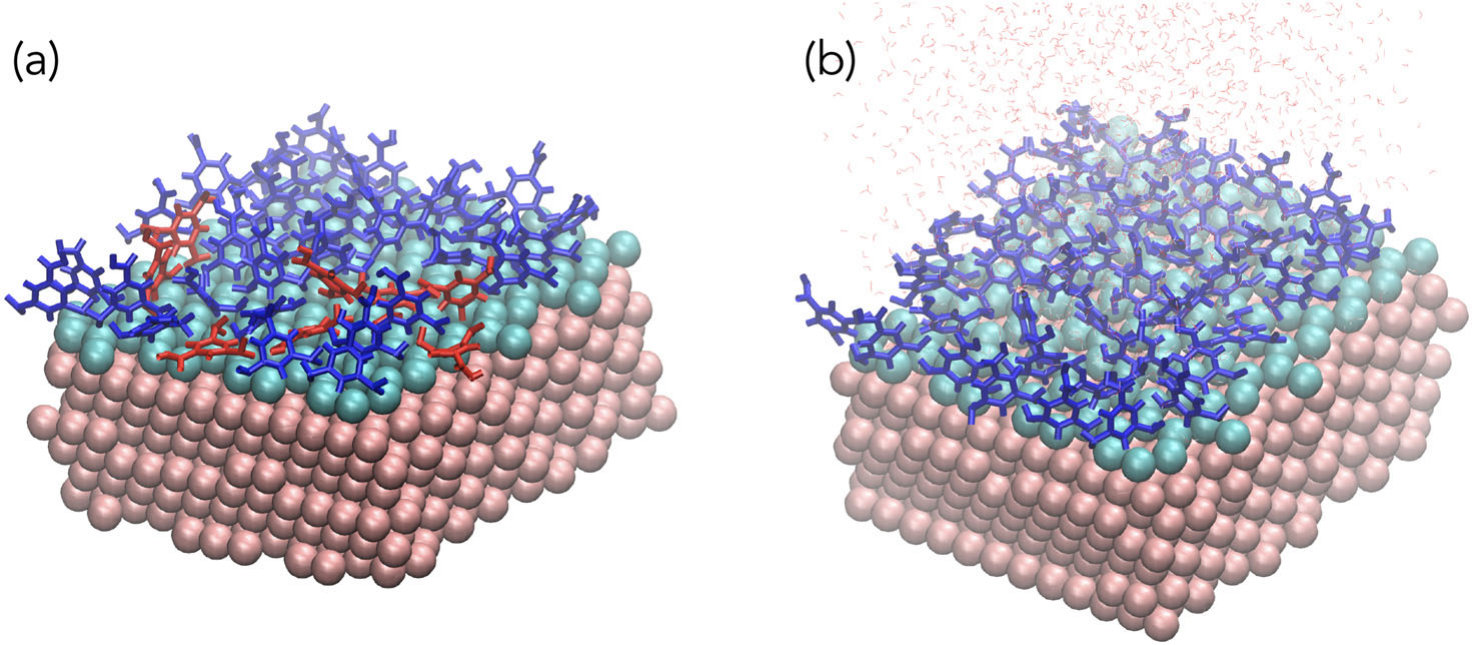
Molecular dynamics simulation snapshots of the TMA monolayer structures on honeycomb Cu/Au(111) (a) in vacuum and (b) in water. For clarity, only a small part of the simulation box is shown. Blue: bipodal adsorption; red: monopodal adsorption.

To understand the adsorption geometry directing effect of the water solvent molecules, we have performed molecular dynamics (MD) simulations that capture the subtle energy balance between TMA–Cu, TMA–TMA and TMA–water interactions governing the resulting geometry. We model TMA adsorption on a honeycomb Cu monolayer [13] on a six‐layer thick Au(111) slab in vacuum and in water to evaluate the stability of two possible adsorption geometries with one or two carboxylate groups bonded to Cu. The last snapshots of the simulated models in vacuum and in water are shown in Figure 2.

The total adsorption energy of the ($H_{2}O$)TMA/Cu system can, in a first approximation, be determined by the number of COO–Cu bonds as well as by the number of hydrogen bonds between TMA and TMA and/or water and the respective bond formation energies. Catlow and coworkers calculated the carboxyl deprotonation and Cu adsorption energy to amount to −0.28 eV per carboxylate linker [35]. Note that the subtle energy differences as estimated from the TERS results above for monopodal and bipodal carboxylate adsorption amount to ca 2.5 meV. This energy difference is too small to be taken into account in the MD. From literature, hydrogen bond formation is typically known to result in an energy gain of −0.24 eV per bond [36].

Our MD results estimate the number of TMA–TMA hydrogen bonds to be about a factor two higher in the monopodal case (ca. 0.96 H‐bonds per TMA molecule) than in the bipodal adsorption geometry (ca. 0.50 H‐bonds per TMA molecule) in vacuum. With respect to the experiment, this result suggest that about 2/3 of the OH stretch TER scattering intensity observed experimentally in Ar is due to TMA in a monopodal adsorption configuration, while bipodally adsorbed TMA only contributes about 1/3 to this TER signal intensity. In vacuum, monopodal or bipodal adsorption geometries do not differ significantly in their total adsorption energies of −0.510 and −0.680 eV, respectively, that is, by only 170 meV/molecule, suggesting that the coexistence of both species at room temperature or slightly elevated temperature in the nearfield is possible.

When adding $H_{2}O$ to the system, the energy balance changes due to the possibility to form hydrogen bonds between TMA and water molecules. Here, interestingly, the average number of TMA–$H_{2}O$ bonds is very similar for the monopodal and bipodal cases, namely around 3.00 and 3.56, respectively. TMA‐TMA interactions are still present in the case of a monopodal COO‐Cu bond (0.87 TMA‐TMA H‐bonds on average per molecule), but negligible (0.23) for the bipodal conformation. In water, the adsorption energies amount to −1.208 eV for one‐leg down and to −1.470 eV for two‐legs down TMA, or a difference of 262 meV/molecule, that is, roughly 10 times kT. This energy barrier for a re‐orientation is much harder to overcome under the given experimental conditions than the one in gas phase, that is, MD indicates a clear thermodynamic preference for bipodal TMA/Cu adsorption in $H_{2}O$, in line with the above discussed TERS and DFT results. Interestingly, the respective numbers of H‐bonds in vacuum (0.5×0.96+0.5×0.5, 50:50 mono/bipodal mixture) and in water (1×3.56, only bipodal) result in a H‐bond ratio of 5.1 water/vacuum, in good quantitative agreement with the experimentally observed ratio of 6.5, as discussed above.

Note that we cannot entirely rule out perturbations of the system under study caused by the presence of the tip and the nearfield, as in any surface‐confined experiment where a large probe apex is located at nm‐distance from a substrate electrode. However, to avoid bias‐induced artefacts, we have ensured that the STM sample bias in all experiments is consistently set to 100 mV, for both in‐Ar and in‐water experiments. Thus, the effect of the sample bias should be identical in both cases and cannot account for the observed spectral differences. Local heating effects due in tip‐sample gaps with field enhancements on the order of 20–30 that are comparable to these of our TERS system, have been reported to be below 10 K in gaseous environment [37]. In water, due to fast heat dissipation, the heating effect can be expected to be lower. Our interpretation relies on the combined analysis of TERS experiments with DFT and MD simulations in which no probe‐induced artefacts are present. Both TERS/DFT and MD results consistently point to a mixed orientation in Ar and a preferential bipodal orientation in water, with a much lower—and thus easier to overcome—energy difference for inert atmosphere compared to aqueous environment.

**FIGURE 3:**
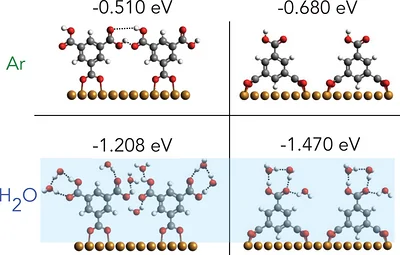
Adsorption model of TMA on honeycomb Cu/Au(111) illustrating the geometry directing character (monopodal or bipodal) of the H─bonds. Adsorption energies obtained from MD as indicated.

In summary, with the help of advanced TERS nanoscopy, DFT and MD, we have resolved the showcase controversy about TMA adsorption geometries at Cu substrates for gas‐phase and water environments. The combined experimental and simulation approach has allowed us to directly compare gas‐ and condensed‐phase conditions and pinpoint similarities and differences with identical sample preparation and measurement scenarios. Our results consistently prove that in gas phase, both monopodal and bipodal adsorption geometries are present while the formation of H‐bonds with water solvent molecules energetically favours bipodal orientation by around 10 kT (Figure 3) at the solid/liquid interface.

Our work highlights the importance of investigating surface behaviour in situ, because gas‐phase approximations are not easily transferable to condensed‐phase environments—which, however, widely dominate sample preparation or reaction conditions. In the future, such precise molecular level information about surface anchoring of organic molecules can be correlated with geometry‐related activity, like molecular self assembly, charge‐transfer efficiency, reaction mechanisms or catalytic conversion, and thus provides an important stepping stone for improved device design.

## Conflicts of Interest

The authors declare no conflicts of interest.

## Supporting information


**Supporting File**: anie73815‐sup‐0001‐SuppMat.pdf.

## Data Availability

All IGOR files and raw data of this TMA/Cu‐TERS project can be made available to readers upon reasonable request.

## References

[1] P. Liu , R. Qin , G. Fu , and N. Zheng , “Surface Coordination Chemistry of Metal Nanomaterials,” Journal of the American Chemical Society 139 (2017): 2122–2131. 10.1021/jacs.6b10978 28085260

[2] Muñoz‐Santiburcio , M. Farnesi Camellone , and D. Marx , “Solvation‐Induced Changes in the Mechanism of Alcohol Oxidation at Gold/Titania Nanocatalysts in the Aqueous Phase Versus Gas Phase,” Angewandte Chemie International Edition 57 (2018): 3327–3331. 10.1002/anie.201710791 29323447

[3] L. Zhang and J. M. Cole , “Anchoring Groups for Dye‐Sensitized Solar Cells,” Applied Materials & Interfaces 7 (2015): 3427–3455. 10.1021/am507334m 25594514

[4] K. L. Materna , R. H. Crabtree , and G. W. Brudvig , “Anchoring Groups for Photovoltaic Water Oxidation on Metal Oxide Surfaces,” Chemical Society Reviews 46 (2017): 6099. 10.1039/c7cs00314e 28640299

[5] E. Leary , A. La Rosa , M. T. González , G. Rubio‐Bollinger , N. Agraït , and N. Martín , “Incorporating Single Molecules Into Electrical Circuits. The Role of the Chemical Anchoring Group,” Chemical Society Reviews 44 (2015): 920. 10.1039/c4cs00264d 25522058

[6] K. Ariga , M. Nishikawa , T. Mori , J. Takeya , L. K. Shrestha , and J. P. Hill , “Self‐Assembly as Key Player for Materials Nanoarchitectonics,” Science and Technology of Advanced Materials 20 (2019): 51–95. 10.1080/14686996.2018.1553108 PMC6374972 30787960

[7] C. Tang , S. Zou , M. W. Severson , and M. J. Weaver , “Coverage‐Dependent Infrared Spectroscopy of Carbon Monoxide on Iridium(111) in Aqueous Solution: A Benchmark Comparison Between Chemisorption in Ordered Electrochemical and Ultrahigh‐Vacuum Environments,” Journal of Physical Chemistry B 102 (1998): 8796–8806.

[8] M. J. Weaver , “Binding Sites and Vibrational Frequencies for Dilute Carbon Monoxide and Nitric Oxide Adlayers in Electrochemical Versus Ultrahigh‐Vacuum Environments: The Roles of Double‐Layer Solvation,” Surface Science 437 (1999): 215–230.

[9] A. Dmitriev , N. Lin , J. Weckesser , J. V. Barth , and K. Kern , “Supramolecular Assemblies of Trimesic Acid on a Cu(100) Surface,” Journal of Physical Chemistry B 106 (2002): 6907–6912.

[10] C. Shen , I. Cebula , C. Brown , J. Zhao , M. Zharnikov , and M. Buck , “Structure of Isophthalic Acid Based Monolayers and Its Relation to the Initial Stages of Growth of Metal‐Organic Coordination Layers,” Chemical Science 3 (2012): 1858–1865.

[11] I. Cebula , H. Lu , M. Zharnikov , and M. Buck , “Monolayers of Trimesic and Isophthalic Acid on Cu and Ag: The Influence of Coordination Strength on Adsorption Geometry,” Chemical Science 4, no. 12 (2013): 4455–4464.

[12] Y. Kim , K. Cho , K. Lee , J. Choo , M. seon Gong , and S. W. Joo , “Electric Field‐Induced Adsorption Change of 1,3,5‐benzenetricarboxylic Acid on Gold, Silver, and Copper Electrode Surfaces Investigated by Surface‐Enhanced Raman Scattering,” Journal of Molecular Structure 878, no. 1‐3 (2008): 155–161.

[13] I. Cebula , C. Shen , and M. Buck , “Isophthalic Acid: A Basis for Highly Ordered Monolayers,” Angewandte Chemie International Edition 49 (2010): 6220–6223. 10.1002/anie.201002082 20632424

[14] P. Verma , “Tip‐Enhanced Raman Spectroscopy: Technique and Recent Advances,” Chemical Reviews 117 (2017): 6447–6466. 10.1021/acs.chemrev.6b00821 28459149

[15] J. H. K. Pfisterer and K. F. Domke , “Unfolding the Versatile Potential of EC‐TERS for Electrocatalysis,” Current Opinion in Electrochemistry 8 (2018): 96–102.

[16] N. J. Jiang , N. Chiang , L. R. Madison , et al., “Nanoscale Chemical Imaging of a Dynamic Molecular Phase Boundary With Ultrahigh Vacuum Tip‐Enhanced Raman Spectroscopy,” Nano Letters 16, no. 6 (2016): 3898–3904. 10.1021/acs.nanolett.6b01405 27183322

[17] N. M. Sabanés , T. Ohto , D. Andrienko , Y. Nagata , and K. F. Domke , “Electrochemical TERS Elucidates Potential‐Induced Molecular Reorientation of Adenine/Au(111),” Angewandte Chemie International Edition 56 (2017): 9796–9801. 10.1002/anie.201704460 28643433

[18] L. Delle Site , C. F. Abrams , A. Alavi , and K. Kremer , “Polymers Near Metal Surfaces: Selective Adsorption and Global Conformations,” Physical Review Letters 89 (2002): 156103. 10.1103/PhysRevLett.89.156103 12366003

[19] D. Andrienko , S. León , L. Delle Site , and K. Kremer , “Adhesion of Polycarbonate Blends on a Nickel Surface,” Macromolecules 38, no. 13 (2005): 5810–5816.

[20] R. Pool , P. Schapotschnikow , and T. J. H. Vlugt , “Solvent Effects in the Adsorption of Alkyl Thiols on Gold Structures: A Molecular Simulation Study,” Journal of Physical Chemistry C 111 (2007): 10201–10212.

[21] E. Mosconi , A. Selloni , and F. De Angelis , “Solvent Effects on the Adsorption Geometry and Electronic Structure of Dye‐Sensitized ${\mathrm{TiO}}_{2}:$ A First‐Principles Investigation,” Journal of Physical Chemistry C 116 (2012): 5932–5940.

[22] A. Byrne , N. J. English , Schwingenschlögl , and D. F. Coker , “Dispersion and Solvent Effects on the Structure and Dynamics of N719 Adsorbed to Anatase Titania (101) Surfaces in Room‐Temperature Ionic Liquids: An *Ab Initio* Molecular Simulation Study,” Journal of Physical Chemistry C 120 (2016): 21–30.

[23] T. Balankura and K. A. Fichthorn , “Solvent Effects on Molecular Adsorption on Ag Surfaces: Polyvinylpyrrolidone Oligomers,” Journal of Physical Chemistry C 122 (2018): 14566–14573.

[24] K.‐Q. Lin , J. Yi , J.‐H. Zhong , et al., “Plasmonic Photoluminescence for Recovering Native Chemical Information From Surface‐Enhanced Raman Scattering,” Nature Communications 8 (2017): 14891, http://www.nature.com/doifinder/10.1038/ncomms14891. 10.1038/ncomms14891 PMC5379060 28348368

[25] F. Latorre , S. Kupfer , T. Bocklitz , D. Kinzel , S. Trautmann , S. Gräfe , and V. Deckert , “Spatial Resolution of Tip‐Enhanced Ramanspectroscopy — DFT Assessment of the Chemical Effect,” Nanoscale 8 (2016): 10229. 10.1039/c6nr00093b 27123952

[26] L. Xiao and Z. D. Schultz , “Spectroscopic Imaging at the Nanoscale: Technologies and Recent Applications,” Analytical Chemistry 90 (2018): 440–458. 10.1021/acs.analchem.7b04151 PMC5750079 29028297

[27] N. Martín Sabanés , L. M. A. Driessen , and K. F. Domke , “Versatile Side‐Illumination Geometry for Tip‐Enhanced Raman Spectroscopy at Solid/Liquid Interfaces,” Analytical Chemistry 88 (2016): 7108–7114. 10.1021/acs.analchem.6b01080 27299508

[28] A. L. Demming , F. Festy , and D. Richards , “Plasmon Resonances on Metal Tips: Understanding Tip‐Enhanced Raman Scattering,” Journal of Chemical Physics 122 (2005): 184716. 10.1063/1.1896356 15918756

[29] J. Li , W. Cao , Y. Shu , et al., “Water Molecules Bonded to the Carboxylate Groups at the Inorganic–Organic Interface of an Inorganic Nanocrystal Coated With Alkanoate Ligands,” National Science Review 9, no. 2 (2021): nwab138, 10.1093/nsr/nwab138. PMC8882163 35233287

[30] L.‐Y. H. Hu , X.‐X. Huang , R.‐Y. Zhou , W. Yao‐Hui , Z.‐L. Yang , and J.‐F. Li , “Experimental Characterization Technique to Probe Interfacial Water,” Journal of Catalysis 430 (2024): 115355.

[31] Y. Chen , S. Zou , K. Huang , and Z. Tian , “SERS Studies of Electrode/Electrolyte Interfacial Water Part II – Librations of Water Correlated to Hydrogen Evolution Reaction,” Journal of Raman Spectroscopy 29 (1998): 749–756.

[32] D.‐Y. Wu , S. Duan , X.‐M. Liu , et al., “Theoretical Study of Binding Interactions and Vibrational Raman Spectra of Water in Hydrogen‐Bonded anionic complexes: ($H_{2}O$) (n = 2 and 3), $H_{2}O$—$X^{-}$ (X = F, Cl, Br, and I), and $H_{2}O$—$M^{-}$ (M = Cu, Ag, and Au),” Journal of Physical Chemistry A 112 (2008): 1313–1321. 10.1021/jp0722105 18215023

[33] M. S. C. Flett , “The Characteristic Infra‐Red Frequencies of the Carboxilic Acid Group,” Journal of the Chemical Society 154 (1951): 962–967.

[34] O. Prakash and R. K. Singh , “Probing Self‐Associated Intermolecular H─Bonding Using Low‐Frequency SERS Coupled With Mid‐IR SERS and DFT Study: A Case Study of 2‐MBA Adsorbed on ZnO Nanoparticles,” Physical Chemistry Chemical Physics 21 (2019): 21431. 10.1039/c9cp03124c 31531501

[35] A. Chutia , I. P. Silverwood , M. Farrow , et al., “Adsorption of Formate Species on Cu(*h,k,l*) Low Index Surfaces,” Surface Science 653 (2016): 45–54.

[36] S. J. Suresh and V. M. Naik , “Hydrogen Bond Thermodynamic Properties of Water From Dielectric Constant Data,” Journal of Chemical Physics 113, no. 21 (2000): 9727–9732. 10.1063/1.3626800 21895225

[37] A. Downes , D. Salter , and A. Elfick , “Heating Effects in Tip‐Enhanced Optical Microscopy,” Optics Express 14 (2006): 5216–5222. 10.1364/oe.14.005216 19516687

