# Supplementary material for: Solvent Hydrogen Bonding Determines Adsorption Geometry of Trimesic Acid on Cu
Source: Angew Chem Int Ed Engl. 2026 Jul 29;65(39):e3523377. doi: 10.1002/anie.3523377 (PMC13592304; doi:10.1002/anie.3523377)
Supplement: Supplementary file 1 [file ANIE-65-e3523377-s001.pdf]

Supporting information: Solvent hydrogen bonding determines adsorption geometry of trimesic acid on Cu

Anusha Lalitha,* Qixuan Li,*

Marialore Sulpizi, Denis Andrienko, and Katrin F. Domke

these authors contributed equally

E-mail: katrin.domke@uni-due.de

Tip-enhanced Raman spectroscopy (TERS)

Our TERS setup consists of a HeNe laser (LSRP-3501, REO, 632.8 nm), scanning tunneling microscope (STM 5420, Keysight Technologies GmbH) and spectrograph (iHR 550, Horiba) with CCD camera (Symphony II, Horiba) connected with optics (Thorlabs GmbH, Germany). Au tips were etched electrochemically as described by Ren et al.[1] and coated with Zapon lack (CLOU, Germany).[2] The TERS setup and tip preparation are described in detail elsewhere.[2]

Sample preparation

Benzene-1,3,5-tricarboxylic acid (TMA; Alfa Aesar, 98%), Cu(II) sulfate pentahydrate (Aldrich), sulfuric acid (Merck, Suprapur, 96%) and absolute ethanol (Sigma Aldrich, 99.9%) were used as received. Ultrapure water (resistivity >18 MΩ/cm) was taken from a Merck MilliQ Integral system. Before every experiment glassware, PCTFE parts and PTFE parts were boiled once in 30% nitric acid and three times in ultrapure water.

TERS substrates were prepared by cleaving mica sheets (Plano GmbH, Germany) and evaporating 150 nm Au on top (Edwards FL400 Auto 306 thermal evaporator). Au/mica substrates were flame annealed for 6 minutes, cleaned with ultrapure water and dried in an oven before copper under potential Cu deposition (CuUPD). CuUPD on top of the Au/mica substrates was performed as described by Cebula et al.[3] Afterwards, the samples were immersed in 1 mM TMA/water solution for 10 minutes, thoroughly cleaned with ethanol and dried with nitrogen. The CuUPD solution and aqueous TMA solution were degassed with Ar before use.

Spectra acquisition

The data presented in the main text of the manuscript are average data of many experiments to improve the signal-to-noise ratio and to obtain a realistic picture of the two different measurement conditions in Argon (example for individual data sets in Figure SI 1) and in MilliQ water (examples for individual data sets in Figure SI 2). We have successfully investigated seven different samples, at least four different locations of each sample, partially also in mapping function, with a total of six tips, amounting to more than 400 spectra. An experiment denoted successful means i) stable STM tunnelling over min two hours; ii) an intact, unbent tip; iii) a reproducible ˜1000 cm-1 ring breathing mode at a detectable signal-to-noise-ration; iv) clean tip-retraction (examples for individual data sets in Figure SI 3) /-reapproach checks (20 nm to 30 mum) with no SERS/farfield response from tip or sample and no carbonaceous contamination signals. In general, MilliQ water was added to the sample cell after successful in-Ar experiments to carry out in-water experiments.

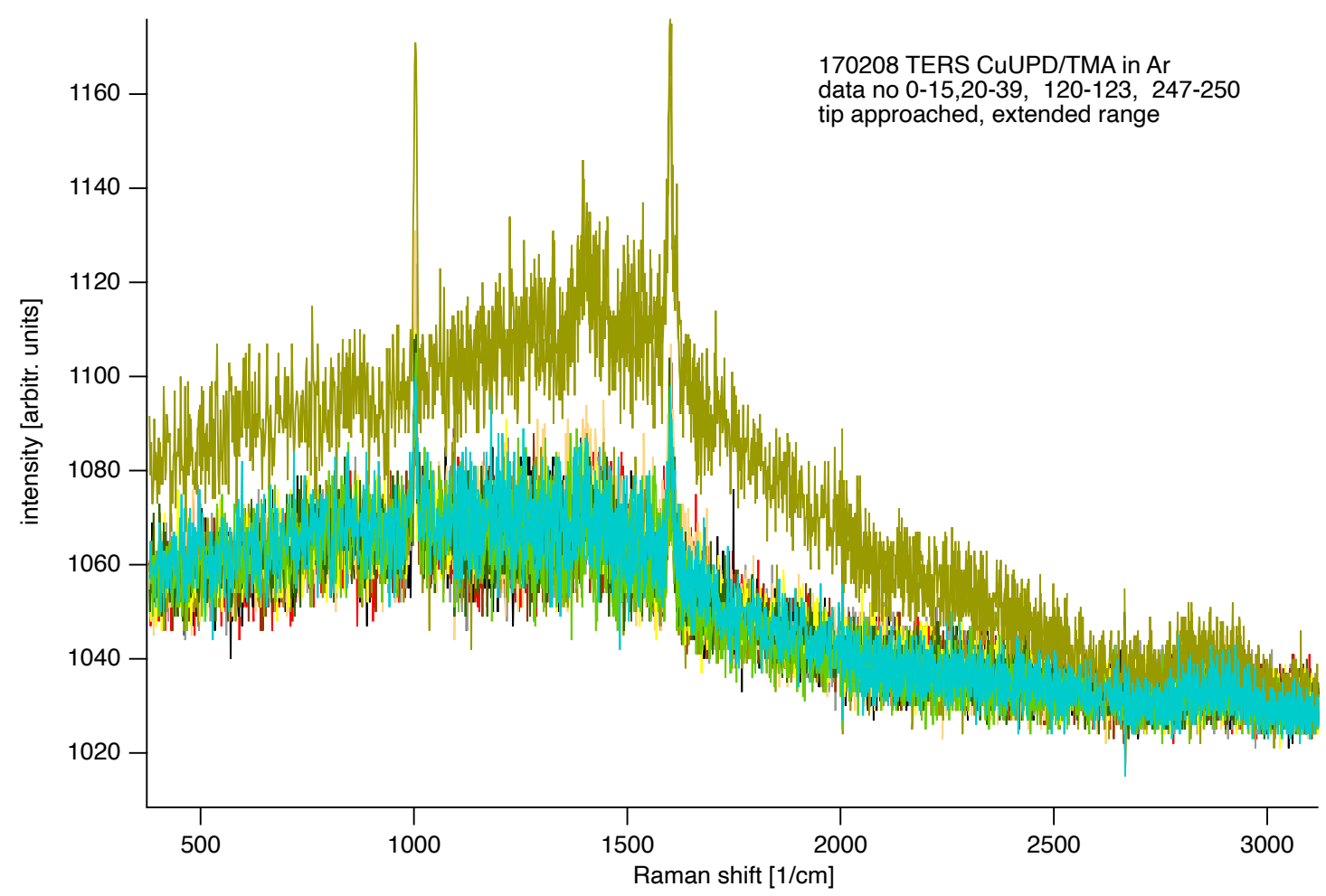

Figure SI 1: Example TERS data sets of TMA on Cu_{UPD}/Au(111) recorded in Ar.

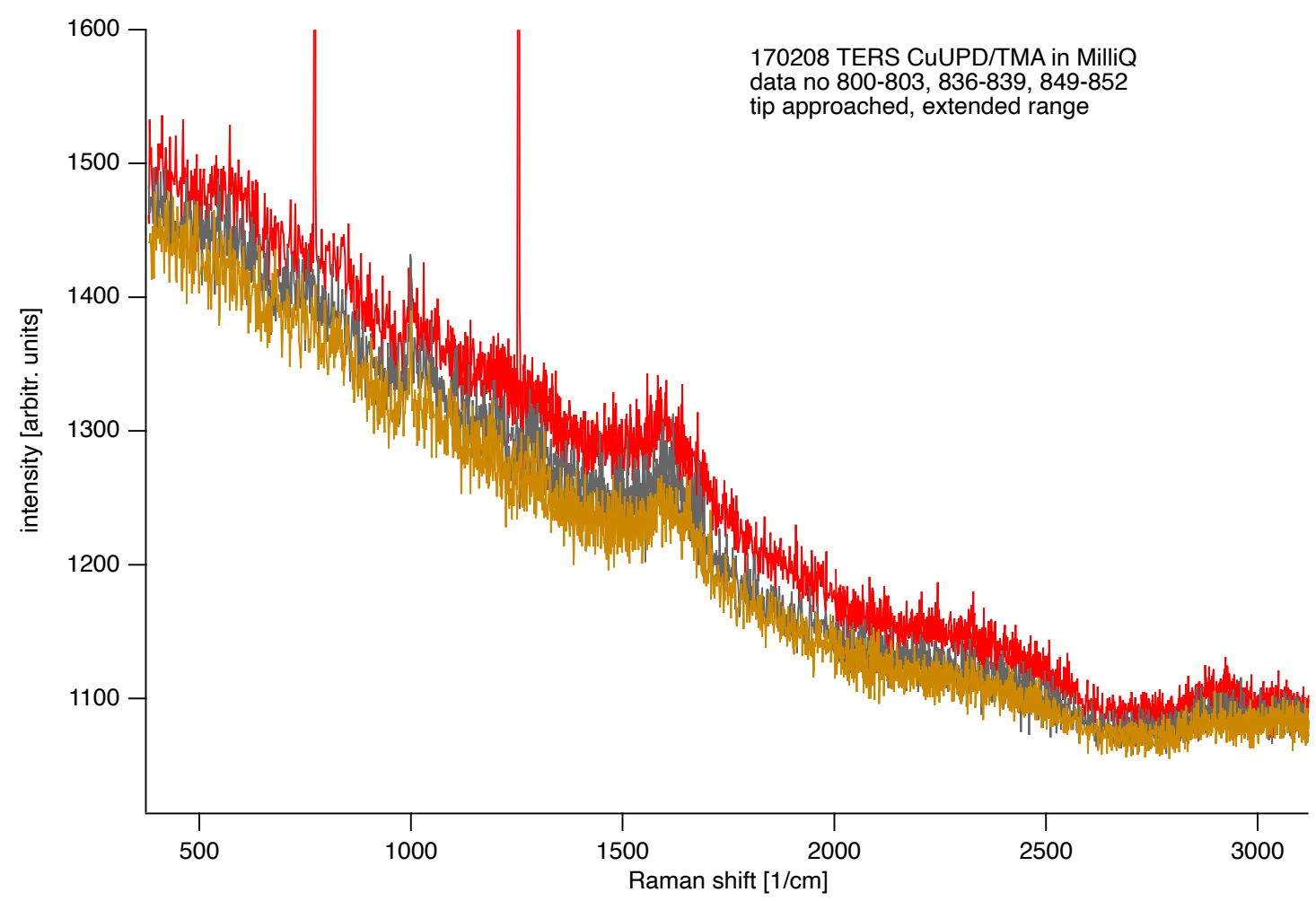

Figure SI 2: Example TERS data sets of TMA on Cu_{UPD}/Au(111) recorded in Milli-Q water.

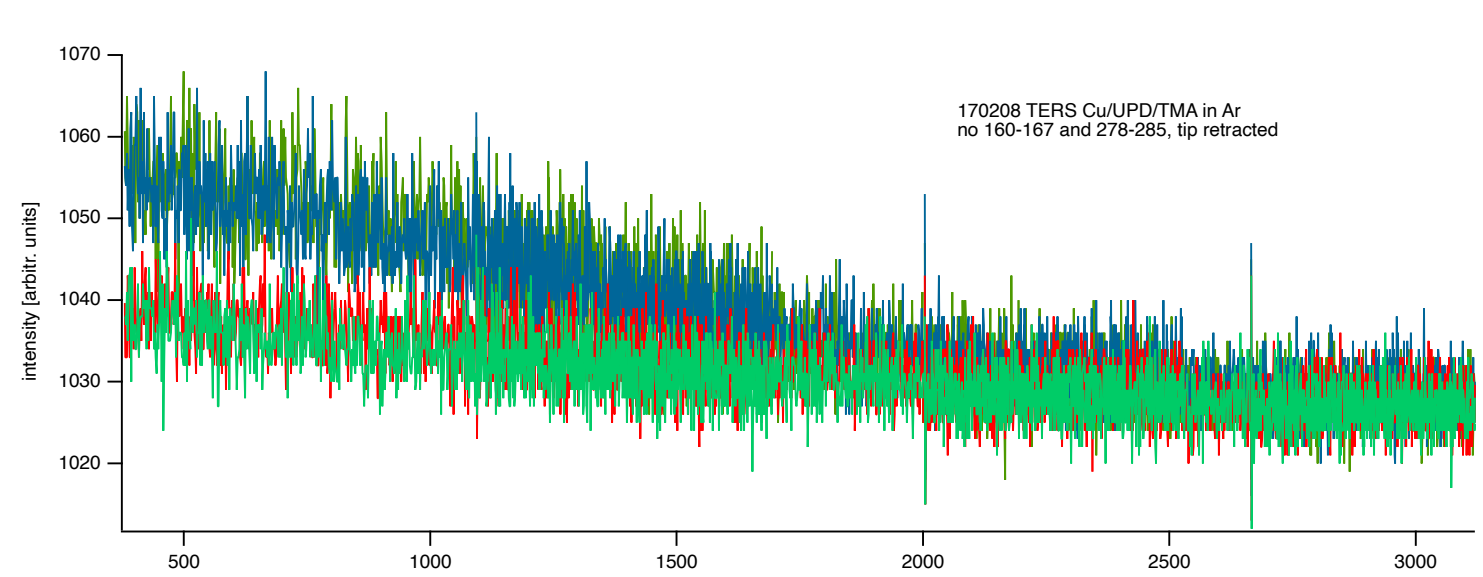

Figure SI 3: Example tip-retraction TERS data sets of TMA on Cu_{UPD}/Au(111) in Ar.

Scanning tunneling microscopy (STM) image before TERS recorded in Argon

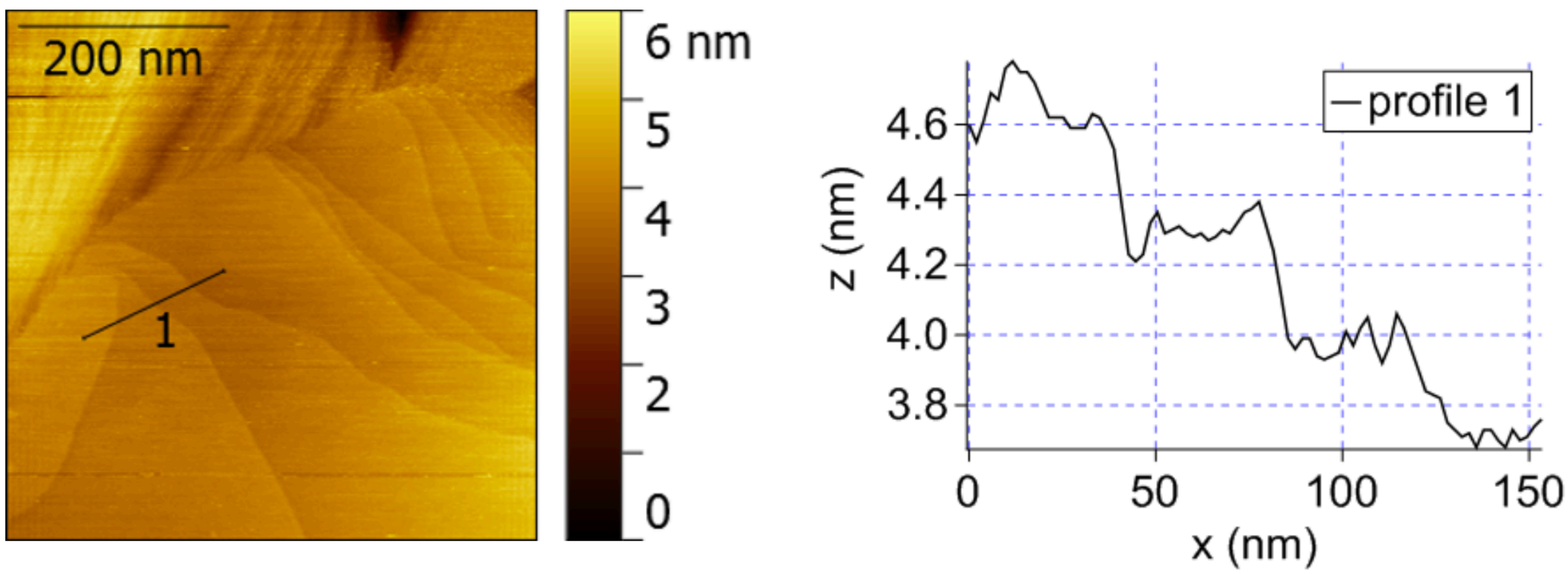

Figure SI 4: Left: STM image of the TMA/Cu_{UPD}/Au(111) substrate recorded in Ar before TER spectra were collected. STM details: Pt/Ir mechanically cut tip, tunneling current setpoint: 10 pA, sample bias voltage: 500 mV, image 500 x 500 nm^2. Right: Height of profile 1 in the STM image on the left.

The STM image in Figure SI 4 of the TMA/Cu_{UPD}/Au(111)-surface recorded in Ar shows atomically flat terraces and monoatomic steps. The line profile indicates a step height of approximately 0.3 nm, slightly larger than the 0.24 nm pure Au(111) step height. For all TERS experiments, the sample surface was first investigated by STM in Ar to assure that TERS experiments were conducted on wide, flat terraces.

TERS peak fitting

We have fitted the region between 350 and 525 cm^{-1} with two Lorentz peaks. The results show that the in-air-spectrum consists of one broad band centered at ca 437 cm^{-1} (Figure SI 5) while the in-water-spectrum depicts two separate modes located at 417 and 480 cm^{-1} (Figure SI 6). Furthermore, we have fitted the region between 2800 and 3120 cm^{-1} with six Lorentz peaks to determine relative band intensities of the OH and the aromatic CH stretch region as well as the full-width-half-maxima of the aromatic CH stretch region for the in-Ar (Figure SI 7) and in-water spectra (Figure SI 8).

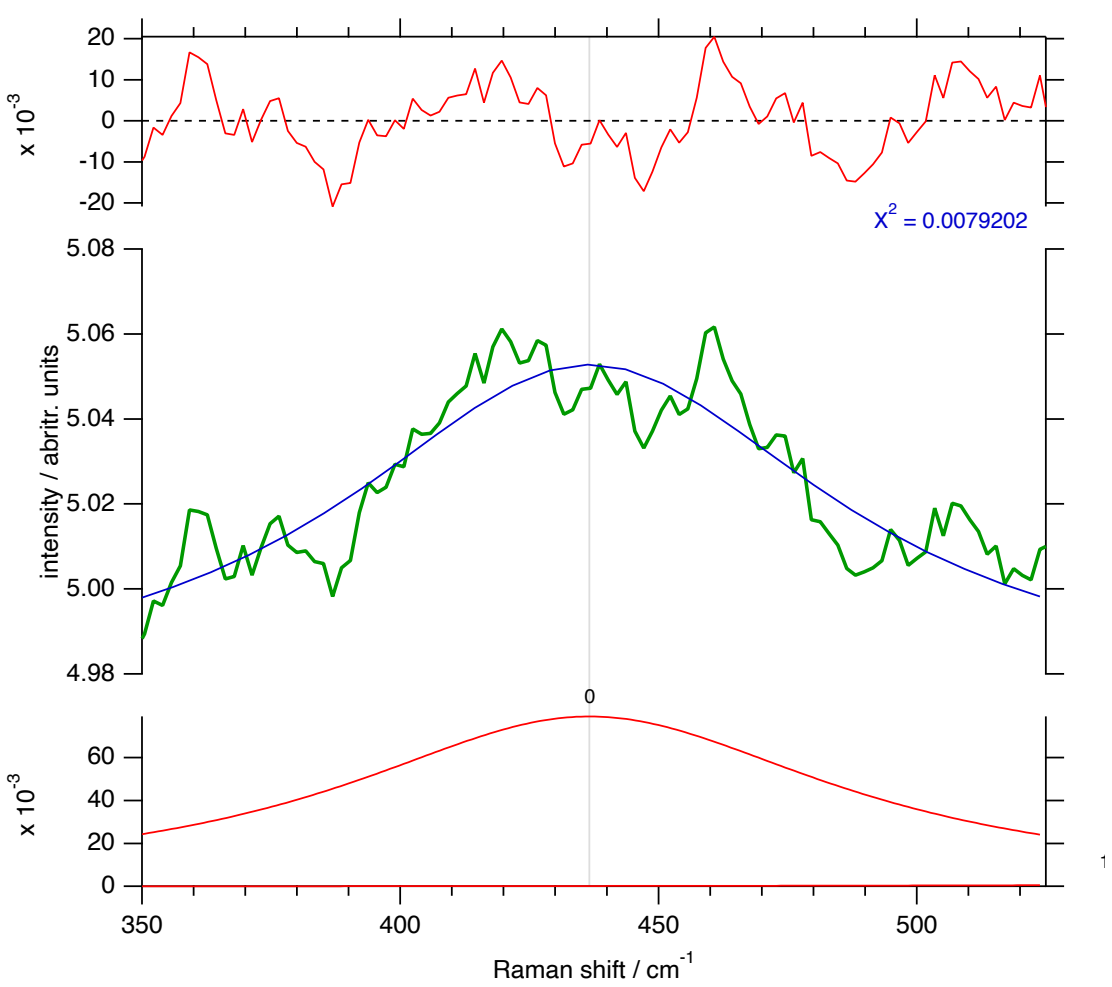

Fit completed: 13:14 Monday, 22. September 2025
Y data wave: root:wave1[169, 271]
X data wave: root:wave0
Chi square: 0.0079202
Total fitted points: 103
Multipeak fit version: 3.00
Total Peak Area = 14.81 +/- 1402

Baseline Type: Constant

y0 =	4.97335	+/- 0.2998

Peak 0 Type: Lorentzian

Location =	436.632	+/- 7.7951
Height =	0.0791446	+/- 0.13593
Area =	14.3966	+/- 35.705
FWHM =	115.803	+/- 89.092

Fit function parameters

Location =	436.632	+/-7.7951
FWHM =	115.803	+/-89.092
Area =	14.3966	+/-35.705

Figure SI 5: Lorentzian peak fitting results of the average TERS spectrum of TMA on Cu_{UPD}/Au(111) in Ar in the region between 350 and 525 cm^{-1}.

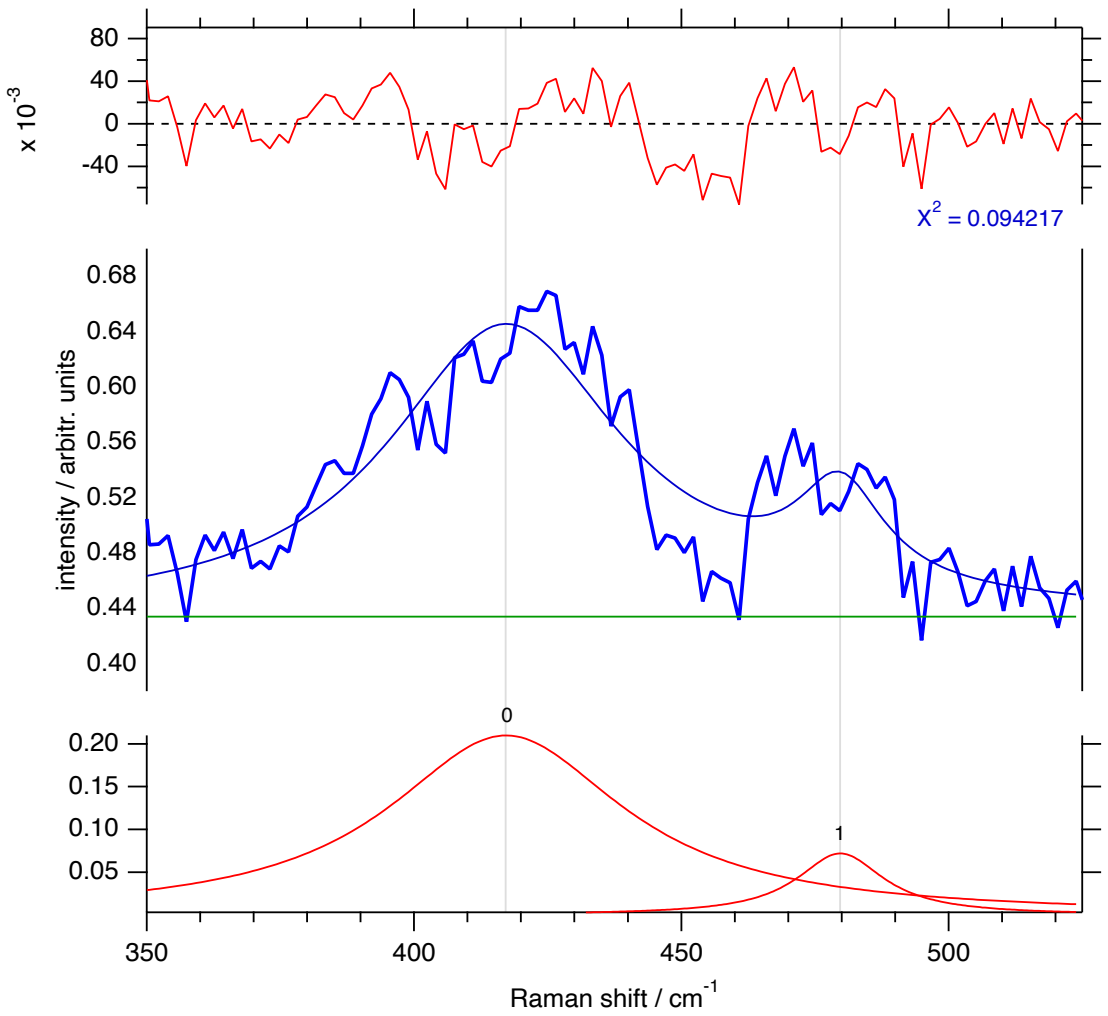

Fit completed: 13:11 Monday, 22. September 2025
Y data wave: root:wave3[169, 271]
X data wave: root:wave2
Chi square: 0.094217
Total fitted points: 103
Multipeak fit version: 3.00
Total Peak Area = 20.005 +/- 2.4089

BaselineType: Constant

y0 =	0.433974	+/- 0.010909

Peak 0 Type: Lorentzian

Location = 417.176	+/- 1.2606
Height = 0.209938	+/- 0.012027
Area = 17.7644	+/- 2.2782
FWHM = 53.869	+/- 5.8544

Fit function parameters

Location =417.176	+/-1.2606
FWHM = 53.869	+/-5.8544
Area = 17.7644	+/-2.2782

Peak 1 Type: Lorentzian

Location = 479.732	+/- 2.1112
Height = 0.071983	+/- 0.015177
Area = 2.24055	+/- 0.7825
FWHM = 19.8155	+/- 7.469

Fit function parameters

Location =479.732	+/-2.1112
FWHM = -19.8155	+/-7.469
Area = 2.24055	+/-0.7825

Figure SI 6: Lorentzian peak fitting results of the average TERS spectrum of TMA on Cu_{UPD}/Au(111) in Milli-Q water in the region between 350 and 525 cm^{-1}.

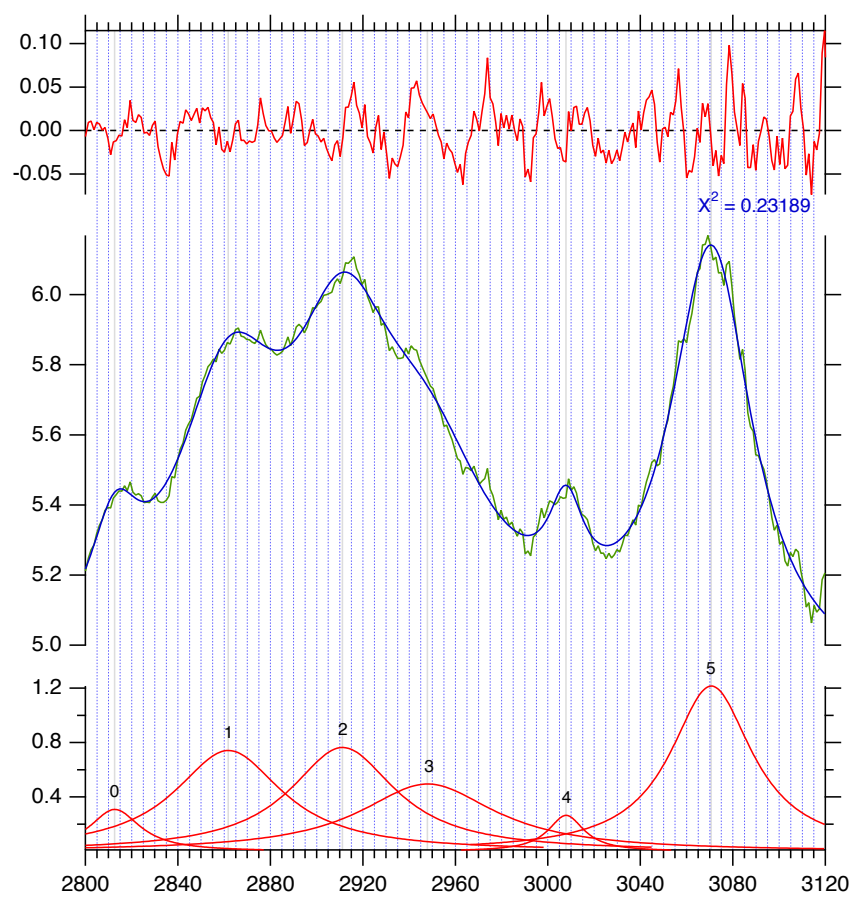

Fit completed: 09:08 Wednesday, 24. September 2025
Y data wave: root:wave1[1863, 2136]
X data wave: root:wave0
Chi square: 0.23189
Total fitted points: 274
Multipeak fit version: 3.00
Total Peak Area = 292.2 +/- 21.457

Baseline Type: Constant

y0 =	4.84517	+/- 0.019589

Peak 0Type: Lorentzian

Location =	2812.69	+/- 0.55838
Height =	0.308987	+/- 0.017906
Area =	13.0105	+/- 1.7057
FWHM =	26.8061	+/- 2.7788
Fit function parameters		
Location =	2812.69	+/-0.55838
FWHM =	26.8061	+/- 2.7788
Area =	13.0105	+/- 1.7057

Peak 1Type: Lorentzian

Location =	2861.82	+/- 0.7326
Height =	0.742289	+/- 0.030119
Area =	66.1508	+/- 6.1303
FWHM =	56.7338	+/- 3.3618
Fit function parameters		
Location =	2861.82	+/- 0.7326
FWHM =	56.7338	+/- 3.3618
Area =	66.1508	+/- 6.1303

Peak 2Type: Lorentzian

Location =	2911.22	+/- 1.1512
Height =	0.764093	+/- 0.091456
Area =	66.7641	+/- 14.858
FWHM =	55.6259	+/- 6.0219
Fit function parameters		
Location =	2911.22	+/- 1.1512
FWHM =	55.6259	+/- 6.0219
Area =	66.7641	+/- 14.858

Peak 3Type: Lorentzian

Location =	2947.98	+/- 3.3997
Height =	0.495806	+/- 0.077415
Area =	55.5755	+/- 13.842
FWHM =	71.3596	+/- 7.488
Fit function parameters		
Location =	2947.98	+/- 3.3997
FWHM =	71.3596	+/- 7.488
Area =	55.5755	+/- 13.842

Peak 4Type: Lorentzian

Location =	3007.87	+/- 0.45444
Height =	0.265391	+/- 0.014566
Area =	7.87873	+/- 0.93297
FWHM =	18.8995	+/- 1.9709
Fit function parameters		
Location =	3007.87	+/-0.45444
FWHM =	18.8995	+/- 1.9709
Area =	7.87873	+/-0.93297

Peak 5Type: Lorentzian

Location =	3070.673	+/- 0.1562
Height =	1.21562	+/- 0.0152
Area =	82.8225	+/- 2.5821
FWHM =	43.3742	+/- 0.972
Fit function parameters		
Location =	3070.673	+/- 0.1562
FWHM =	43.3742	+/- 0.972
Area =	82.8225	+/- 2.5821

Figure SI 7: Lorentzian peak fitting results of the average TERS spectrum of TMA on Cu_{UPD}/Au(111) in Ar water in the region between 2800 and 3120 cm^{-1}.

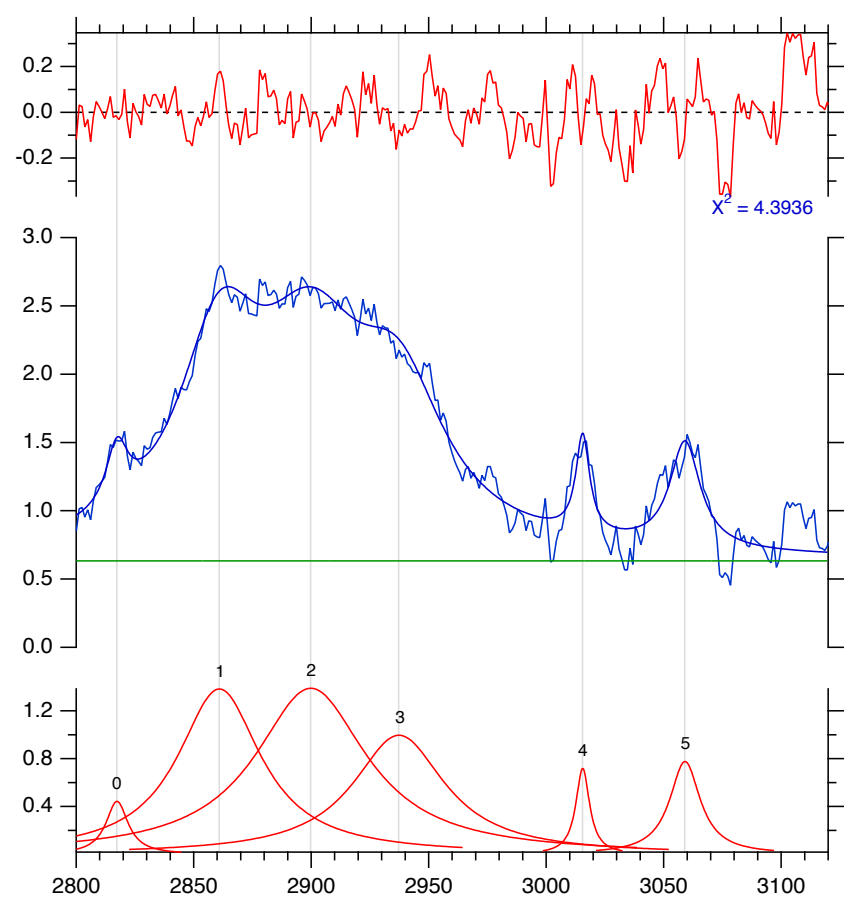

Fit completed: 09:11 Wednesday, 24. September 2025
Y data wave: root:wave3[1865, 2138]
X data wave: root:wave2
Chi square: 4.3936
Total fitted points: 274
Multipeak fit version: 3.00
Total Peak Area = 329.8 +/- 76.35

Baseline Type: Constant

y0 =	0.633198	+/- 0.026813

Peak 0 Type: Lorentzian

Location =	2817.32	+/- 0.89817
Height =	0.443586	+/- 0.070427
Area =	7.9505	+/- 2.0397
FWHM =	11.4103	+/- 3.2001

Fit function parameters

Location =	2817.32	+/-0.89817
FWHM =	11.4103	+/- 3.2001
Area =	7.9505	+/- 2.0397

Peak 1 Type: Lorentzian

Location =	2860.88	+/- 1.625
Height =	1.3838	+/- 0.22646
Area =	93.6701	+/- 26.885
FWHM =	43.0932	+/- 5.9741

Fit function parameters

Location =	2860.88	+/- 1.625
FWHM =	43.0932	+/- 5.9741
Area =	93.6701	+/- 26.885

Peak 2 Type: Lorentzian

Location =	2899.82	+/- 2.2411
Height =	1.38976	+/- 0.28007
Area =	126.139	+/- 63.435
FWHM =	57.782	+/- 17.782

Fit function parameters

Location =	2899.82	+/- 2.2411
FWHM =	57.782	+/- 17.782
Area =	126.139	+/- 63.435

Peak 3 Type: Lorentzian

Location =	2937.31	+/- 2.9679
Height =	0.996742	+/- 0.27032
Area =	74.8248	+/- 32.746
FWHM =	47.7906	+/- 8.6573

Fit function parameters

Location =	2937.31	+/- 2.9679
FWHM =	47.7906	+/- 8.6573
Area =	74.8248	+/- 32.746

Peak 4 Type: Lorentzian

Location =	3015.51	+/- 0.41888
Height =	0.718542	+/- 0.085188
Area =	7.98954	+/- 1.1328
FWHM =	7.07864	+/- 1.2909

Fit function parameters

Location =	3015.51	+/-0.41888
FWHM =	-7.07864	+/- 1.2909
Area =	7.98954	+/- 1.1328

Peak 5 Type: Lorentzian

Location =	3059.04	+/- 0.57772
Height =	0.776333	+/- 0.057553
Area =	19.2302	+/- 2.2002
FWHM =	15.7694	+/- 2.0425

Fit function parameters

Location =	3059.04	+/-0.57772
FWHM =	15.7694	+/- 2.0425
Area =	19.2302	+/- 2.2002

Figure SI 8: Lorentzian peak fitting results of the average TERS spectrum of TMA on Cu_{UPD}/Au(111) in Milli-Q water in the region between 2800 and 3120 cm^{-1}.

Density functional theory (DFT)

Molecules were first optimized using the CAM-B3LYP functional and 6-31g(d,p) basis set using the GAUSSIAN09 package.[4] For metal atoms, we used the built-in Hay-Wadt pseudopotentials supplied with the lanl2dz basis set. The pre-resonance Raman intensities (averaged over molecular orientations) were computed by evaluating polarizability derivatives by numerically differentiating the analytic dipole derivatives with respect to an electric field.

We calculated the orientation-dependent Raman intensity by transferring the molecular polarizability calculated in the molecular frame to that in the lab frame. The results are summarized in Table SI 1 and displayed in Figure SI 9.

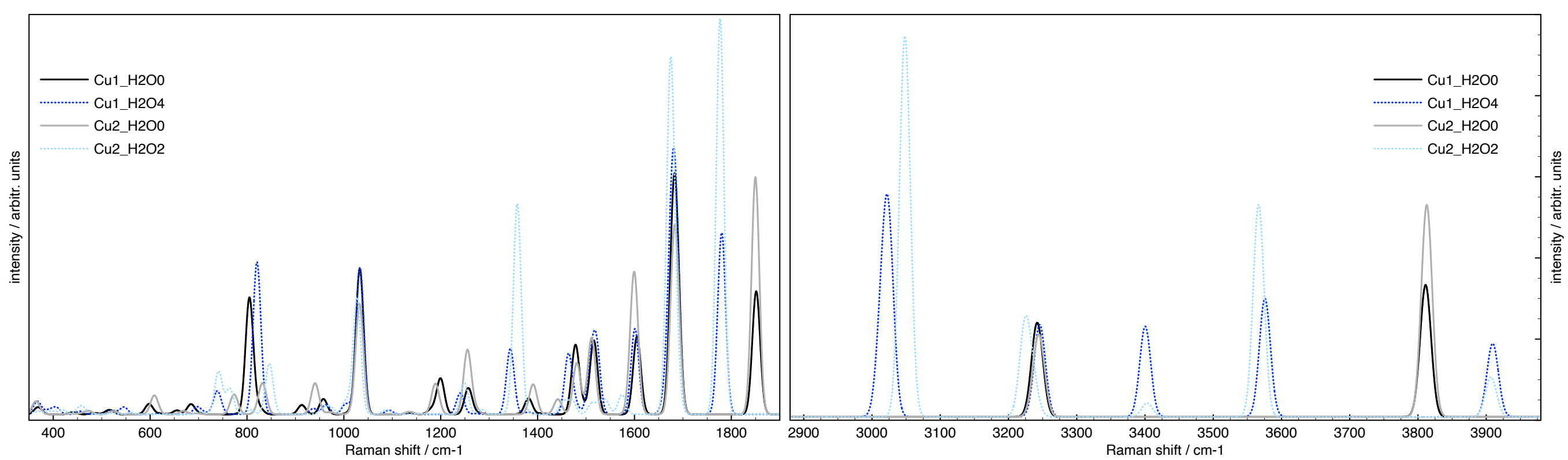

Figure SI 9: Rendering of DFT results with arbitrary line width.

The observed TERS frequencies differ from the ones reported for ensemble surface-enhanced Raman scattering of TMA/Cu by Kim et al.,[5] likely because because of the difference in substrate potential and because the literature spectra are average responses from large molecular ensembles covering a variety of energetically different adsorption sites as opposed to molecules with homogeneous adsorption structure in our report.

Table SI 1: Overview of measured and DFT-calculated Raman frequencies of TMA.

Experiment TERS in Ar	Experiment TERS in H_2O	DFT 1-Cu	2-Cu	1-Cu/H_2O	2-Cu/H_2O	TMA/H_2O	TMA tetramer	assignment
437	417, 480	427	423, 443	441	438, 447			νCuO(COOCu)
841, sh 869	863	886, 929	913 (942)	(909) 934	(929) 944	910	915	νCC (C-COOCu), ring def
1002, sh 1035	999, sh 1035	1002	1002	1002	996	1003	1002	ring breathe
	sh970, sh1027			974, 1060	975, 1054			H_2O bending (H-bonded)
	1093	(1100, 1104)	(1103) 1234	(1235)	1245	1106, 1108	1111	CH_{arom} bend
1146, 1181	1198	1145, 1164	1153	1203 (1235)	1212	1198	1202	νC-OH
1290	1286	1219	1217	1304	1317	1239, 1307, 1308	1243, 1306	COH bend
1403		1340	1349					νC-COOH
1446, 1602	1446, 1592	1434, 1448, 1471, 1557, 1630, 1636	1399, 1437, 1466, 1548, 1553, 1631, 1637	1420, 1465, 1475, 1553, 1635	1409, 1427, 1482, 1494, 1524, 1624	1316, 1441, 1442, 1625, 1632	1317,1430, 1449, 1629, 1630	νCC_{arom}, νCC (C-COOCu)
	sh1560, sh1623			1612, 1627	1615, 1627			H_2O scissor (H-bonded)
1734, 1787	1673	1795, 1797	1795	1725, 1729	1724	1726, 1731	1689, 1741, 1743	νCO (COOH)
2800-3020	2810-3035			2896	2862, 2873	2890, 2891, 2906	2990, 3087, 3118	νOH (H-bonded COOH and network water)
3071	3059	3142, 3145, 3150	3142, 3147, 3150	3145, 3149, 3152	3127, 3134, 3140	3149, 3150	3147	νCH_{arom}

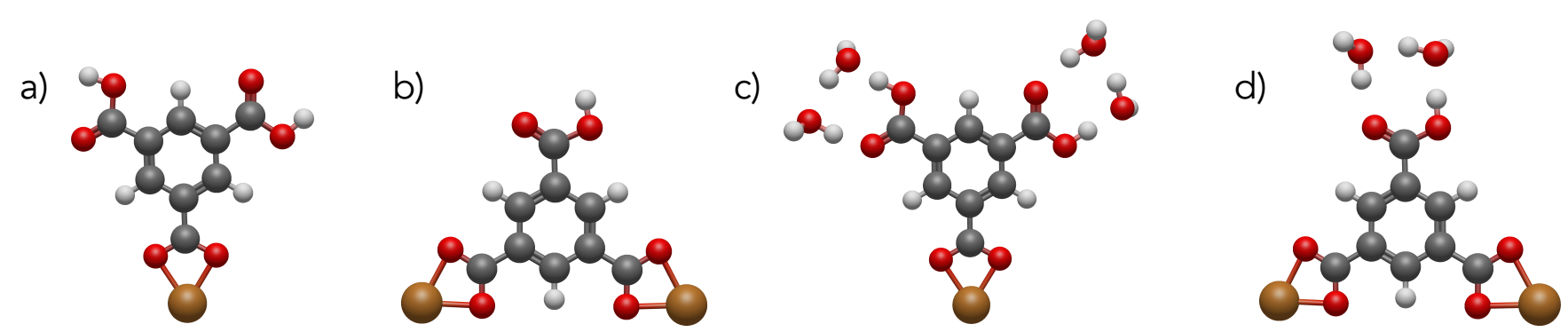

Figure SI 10: Sketches of the different TMA structures used for DFT calculations. a) 1-Cu: TMA bound to Cu with one carboxylate group. b) 2-Cu: TMA bound to Cu with two carboxylate groups. c) 1-Cu/H2O: TMA bound to Cu with one carboxylate group and four hydrogen bonds to neighbouring water molecules. d) 2-Cu/H2O: TMA bound to Cu with two carboxylate groups and two hydrogen bonds to neighbouring water molecules.

Molecular dynamics (MD) simulations

Methods

All molecular dynamics (MD) simulations were performed using GROMACS 2022.1. A monolayer of either a (polarizable) honeycomb Cu structure or a (polarizable) Cu(111) layer was underpotentially deposited (UPD) onto an Au(111) slab consisting of six atomic layers. The hexagonal honeycomb Cu overlayer on Au(111) is illustrated in Figure SI 11 The commensurate relationship between the Cu honeycomb and the Au(111) substrate follows Cai Shen et al.:[6] Accordingly, the honeycomb Cu layer was constructed using the Au(111) lattice constant. This honeycomb monolayer corresponds to a Cu coverage of 2/3 of the first Au(111) layer, consisting of 128 Cu atoms deposited on a surface containing 192 Au atoms.

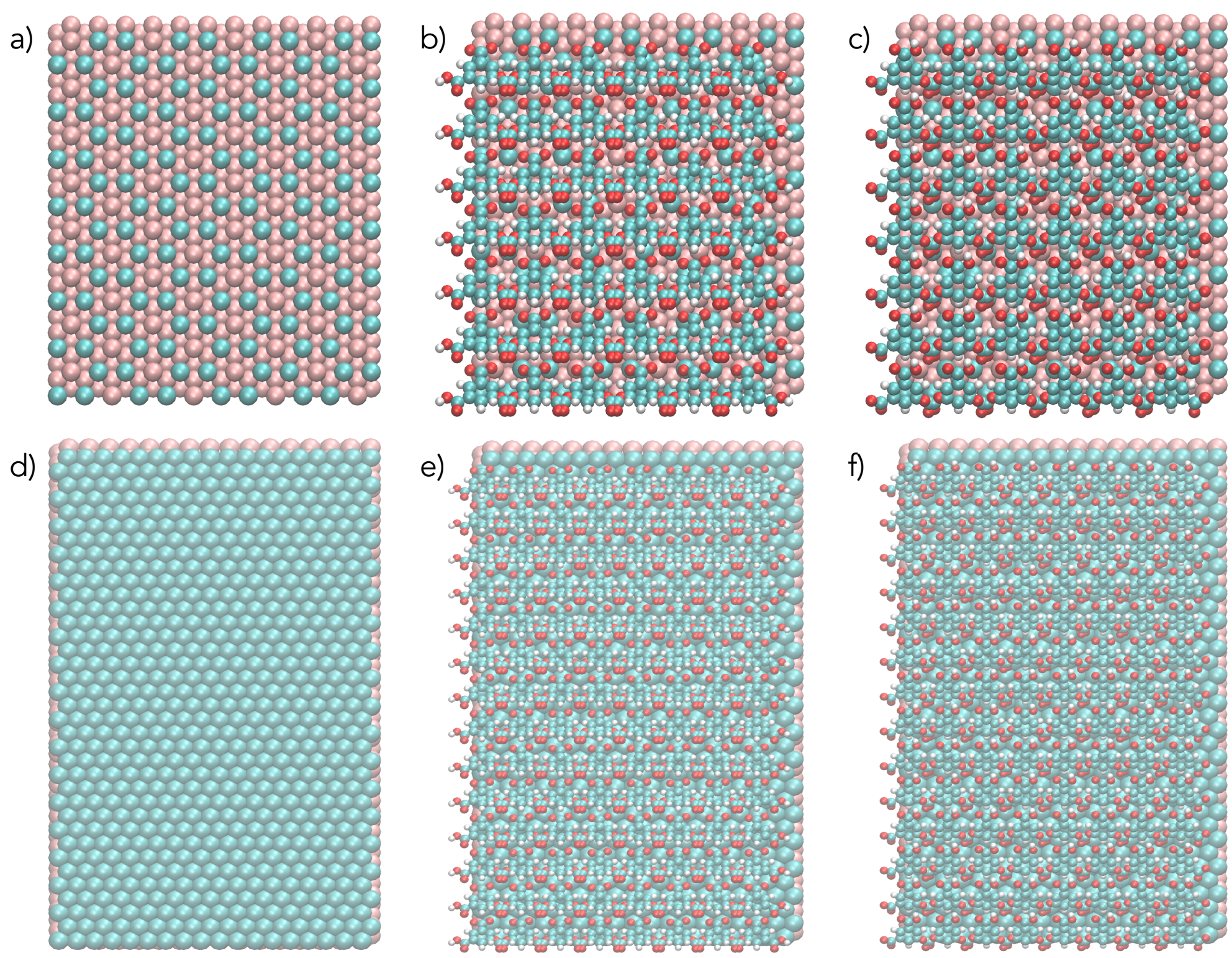

Figure SI 11: Top row: a) Honeycomb Cu adlayer on six layers Au(111) slab with the initial configuration of 42 TMA molecules in a) monopodal and c) bipodal adsorption geometry. Bottom row: d) Cu(111) adlayer on six layers Au(111) with 112 TMA molecules in e) monopodal and f) bipodal adsorption geometry.

For vacuum simulations, the simulation box dimensions were set to $3.46 \times 3.99 \times 4.17$ nm^3 for the honeycomb Cu/Au(111) system and to $4.62 \times 7.99 \times 4.18$ nm^3 for the Cu(111)/Au(111) system, ensuring periodic boundary conditions for both the Cu overlayer and the Au substrate. For the in-liquid simulations, 42 TMA molecules were deposited onto

the honeycomb Cu surface together with 3000 SPC/E water molecules. For the Cu(111) surface, 112 TMA molecules and 10,000 SPC/E water molecules were added. Consequently, the initial simulation box dimensions for the solvated systems were $3.46 \times 3.99 \times 10.17$ nm^3 for the honeycomb Cu/Au(111) system and $4.62 \times 7.99 \times 10.18$ nm^3 for the Cu(111)/Au(111) system. In all initial configurations, the TMA molecules were arranged in rows with their aromatic rings tilted by approximately 45° relative to the surface normal, following the experimental observations reported in by Cai Shen et al.[6]

The GROMOS54a7 force field was employed for all MD simulations, while the SPC/E model was used to represent water molecules. The force-field parameters for TMA were generated using the Automated Topology Builder (ATB) version 3.0, adopting the bonded and van der Waals parameters from the GROMOS force field. The partial atomic charges of TMA were assigned according to previously reported values. Both singly deprotonated (monopodal) and doubly deprotonated (bipodal) TMA molecules were modeled as charge-neutral species. Periodic boundary conditions were applied in all three spatial directions. Prior to production simulations, each system was energy-minimized until the maximum force on any atom was below 100 kJ/(mol nm). Both solvated and vacuum systems were subsequently pre-equilibrated for 400 ps. Production simulations were then carried out for 12 ns using a 0.5 fs integration time step. The final 1 ns of each trajectory was used for hydrogen-bond analysis. The temperature and pressure were maintained at 300 K and 1 bar, respectively, using the velocity-rescale (V-rescale) thermostat and the Parrinello–Rahman barostat. Hydrogen-bond analysis was performed using the gmx hbond module in GROMACS. Hydrogen bonds were identified using a strict geometric criterion, with a hydrogen-donor-acceptor angle of $\leq$ 30° and a donor-acceptor distance of ≤ 0.35 nm.

Results and discussion

After an initial equilibration period of 12 ns, all simulation systems reached stable configurations. The last 10 ns of each trajectory were subsequently used to calculate the water mass density profiles (Figure SI 12. In both the monopodal (1-leg-down) and bipodal (2-leg-down) configurations, TMA molecules remain strongly adsorbed on both the honeycomb Cu and Cu(111) surfaces supported on Au(111). These results indicate that the Cu UPD monolayer predominantly governs the adsorption behavior, while the underlying Au(111) substrate has a negligible direct influence on TMA adsorption.

We observe a higher water density at the Cu interface for the 2-leg-down TMA configuration than for the 1-leg-down TMA configuration. This behavior may arise from stronger interactions between water molecules and the 2-leg-down TMA species. In addition, a larger number of water molecules can penetrate the intermolecular regions between adjacent TMA

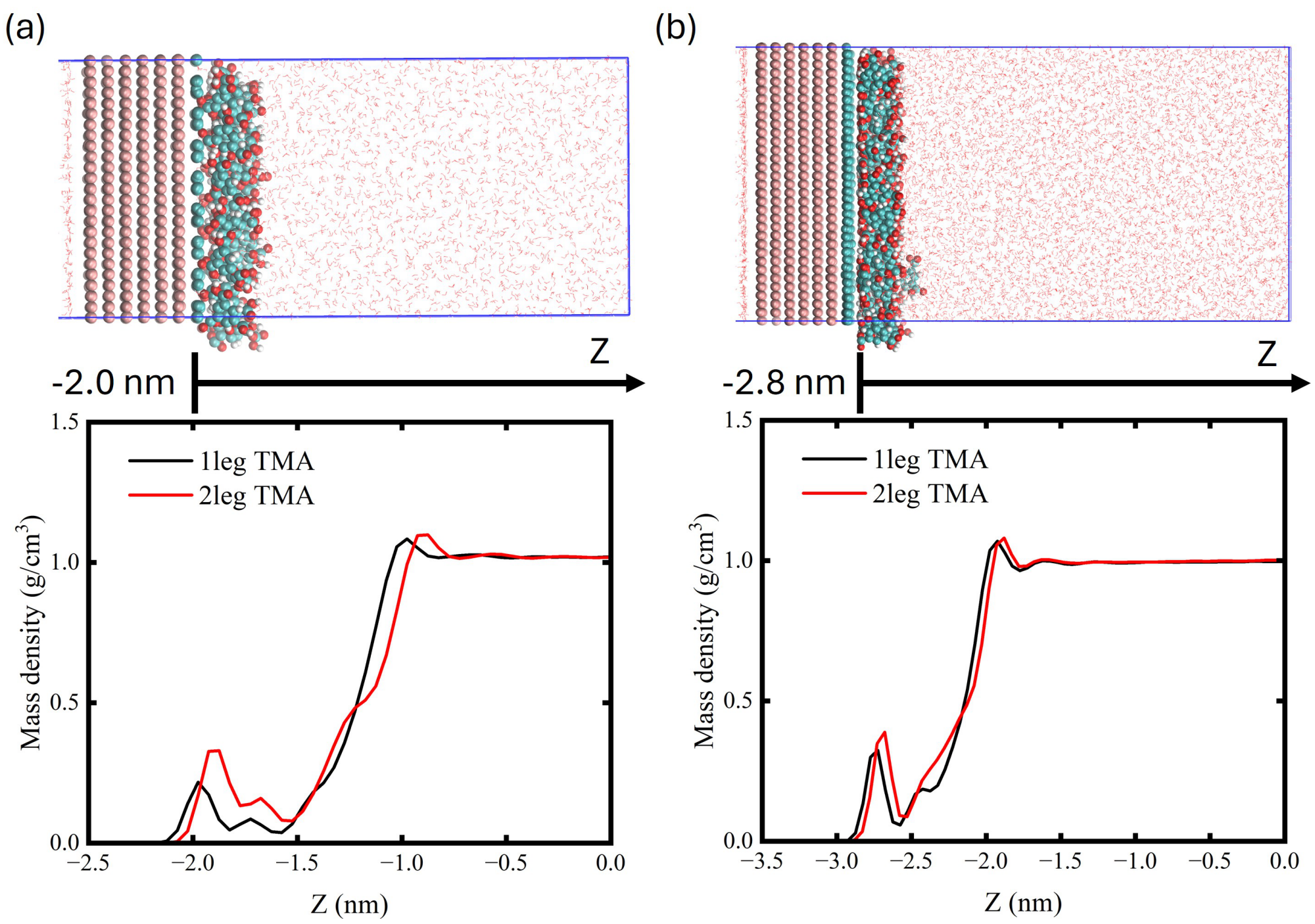

Figure SI 12: The mass density profile of water along z-axis for a) honeycomb Cu and b) Cu(111) on top of 6 layers Au(111).

molecules, thereby increasing the average TMA-TMA separation and weakening the intermolecular TMA-TMA interactions.

Furthermore, a greater accumulation of water is observed at the Cu(111) surface than at the honeycomb Cu surface. This difference may be attributed to the enhanced ability of Cu(111) to stabilize the adsorbed TMA layer, as well as its larger exposed Cu surface area, which provides more adsorption sites for water molecules.

Table SI 2 shows that the bipodal configuration substantially reduces the number of hydrogen bonds between neighboring TMA molecules, while slightly increasing the number of hydrogen bonds formed between TMA and water molecules. Furthermore, for both the monopodal and bipodal configurations, the honeycomb Cu surface exhibits fewer TMA-water hydrogen bonds than the Cu(111) surface. This trend is consistent with the lower interfacial water density observed for the honeycomb Cu surface, which reduces the number of water molecules available to form hydrogen bonds with the adsorbed TMA layer.

The adsorption energies, summarized in Table SI 3 further support these observations. Regardless of whether the simulations were performed in vacuum or in water, the adsorption energies on Cu(111) are consistently more negative than those on honeycomb Cu, indicating that Cu(111) binds TMA more strongly. This finding is consistent with the structural characteristics of the two Cu overlayers: the close-packed Cu(111) surface provides a higher

Table SI 2: The average number of hydrogen-bonds between TMA and water or TMA and TMA in the case of 1-leg down TMA and 2-leg down TMA at the top of honeycomb Cu and Cu(111) in water, and the average number of hydrogen-bonds between TMA and TMA in the case of 1-leg down TMA and 2-leg down TMA at the top of honeycomb Cu and Cu(111) in vacuum.

system	monopodal adsorption		bipodal adsorption	
	TMA-H_2O	TMA-TMA	TMA-H_2O	TMA-TMA
honeycomb Cu/Au(111) in water	3.00 ± 0.06	0.87 ± 0.02	3.56 ± 0.04	0.23 ± 0.05
Cu(111)/Au(111) in water	3.48 ± 0.07	0.84 ± 0.02	3.69 ± 0.04	0.19 ± 0.01
honeycomb Cu/Au(111) in vacuum	/	0.96 ± 0.07	/	0.50 ± 0.03
Cu(111)/Au(111) in vacuum	/	1.28 ± 0.01	/	0.56 ± 0.03

Table SI 3: Summary of the adsorption energies [eV] calculated for the cases of monopodal (1-leg down) TMA and bipodal (2-leg down) TMA on top of honeycomb Cu and of Cu(111) at Au(111) in water and in vacuum.

system	monopodal adsorption	bipodal adsorption
honeycomb Cu/Au(111) in water	-1.208 ± 0.019	-1.470 ± 0.022
Cu(111)/Au(111) in water	-1.317 ± 0.022	-1.491 ± 0.012
honeycomb Cu/Au(111) in vacuum	-0.510 ± 0.017	-0.680 ± 0.007
Cu(111)/Au(111) in vacuum	-0.587 ± 0.002	-0.694 ± 0.007

density of Cu atoms and more favorable adsorption sites than the honeycomb Cu layer, resulting in stronger TMA-surface interactions.

Overall, the honeycomb Cu surface consistently exhibits less negative adsorption energies than Cu(111), indicating weaker stabilization of TMA molecules at the surface. This difference is particularly pronounced for the 1-leg-down TMA configuration in both vacuum and aqueous environments. In contrast, for the 2-leg-down configuration, TMA molecules readily form intermolecular hydrogen bonds in vacuum, resulting in a more strongly bound adsorbed layer.

Furthermore, the Cu(111)/Au(111) system exhibits adsorption energies and average numbers of hydrogen bonds that are comparable to those of the pure Cu(111) slab (six atomic layers), indicating that the underlying Au(111) substrate has little influence on the adsorption behavior once the close-packed Cu overlayer is formed. By comparison, the honeycomb Cu/Au(111) system consistently displays weaker TMA adsorption, highlighting the significant role of the surface Cu atomic arrangement in governing the adsorption strength.

References

(1) Ren, B.; Picardi, G.; Pettinger, B. Preparation of gold tips suitable for tip-enhanced Raman spectroscopy and light emission by electrochemical etching. *Review of Scientific Instruments* **2004**, *75*, 837–841.

(2) Martín Sabanés, N.; Driessen, L. M. A.; Domke, K. F. Versatile Side-Illumination Geometry for Tip-Enhanced Raman Spectroscopy at Solid/Liquid Interfaces. *Analytical Chemistry* **2016**, *88*, 7108–7114.

(3) Cebula, I.; Lu, H.; Zharnikov, M.; Buck, M. Monolayers of trimesic and isophthalic acid on Cu and Ag: the influence of coordination strength on adsorption geometry. *Chemical Science* **2013**, *4*, 4455–4464.

(4) Frisch, M. J. et al. Gaussian09 Revision E.01. Gaussian Inc. Wallingford CT 2009.

(5) Kim, Y.; Cho, K.; Lee, K.; Choo, J.; seon Gong, M.; Joo, S. W. Electric field-induced adsorption change of 1,3,5-benzenetricarboxylic acid on gold, silver, and copper electrode surfaces investigated by surface-enhanced Raman scattering. *Journal of Molecular Structure* **2008**, *878*, 155–161.

(6) Shen, C.; Cebula, I.; Brown, C.; Zhao, J.; Zharnikov, M.; Buck, M. Structure of isophthalic acid based monolayers and its relation to the initial stages of growth of metal-organic coordination layers. *Chemical Science* **2012**, *3*, 1858–1865.
